# Microbial Contamination of Chicken Litter Manure and Antimicrobial Resistance Threat in an Urban Area Setting in Cameroon

**DOI:** 10.3390/antibiotics10010020

**Published:** 2020-12-29

**Authors:** Marie Paule Ngogang, Tambo Ernest, Jennifer Kariuki, Mohamed Moctar Mouliom Mouiche, Jeanne Ngogang, Abel Wade, Marianne Antonia Bernada van der Sande

**Affiliations:** 1 Laboratoire de Recherche et d’Expertise Biomédicale (LABOREB), P.O. Box 35262 Yaoundé, Cameroon; tambo0711@gmail.com (T.E.); jngogang@yahoo.fr (J.N.); 2 Institute of Tropical Medicine, 2000 Antwerp, Belgium; jenykariuki@gmail.com (J.K.); mvandersande@itg.be (M.A.B.v.d.S.); 3 Higher Institute of Health Sciences, Université des Montagnes, P.O. Box 208 Bagangté, Cameroon; 4 School of Veterinary Medicine and Sciences, University of Ngaoundere, P.O. Box 454 Ngaoundere, Cameroon; mouichemoctar4@gmail.com; 5 Laboratoire National Veterinaire (LANAVET), P.O. Box 503 Garoua, Cameroon; abelwade@gmail.com; 6 Global Health, Julius Centre for Health Sciences and Primary Care, University Medical Centre Utrecht, Utrecht University, 3584 CG Utrecht, The Netherlands

**Keywords:** antimicrobial resistance, veterinary antibiotic use, chicken litter manure

## Abstract

We conducted a pilot study to assess microbiological safety of chicken litter, an affordable organic and main fertilizer used in Cameroon and worldwide. A convenience sampling of 26 farms was done and a questionnaire was administered. Samples of litter were aseptically collected. *E. coli* and *Salmonella* spp. were isolated using CLSI standards. Antibiotic susceptibility testing was performed using the disc diffusion method and a micro broth dilution method for colistin. In broiler farms, 90% of participating farmers gave antibiotic prophylaxis. The prevalence of *E. coli* and *Salmonella* spp. was 59.1% and 15.5%, respectively. All *E. coli* isolates were multidrug resistant as well as 36.4% for *Salmonella* spp. No resistance was found against cefepime and imipenem. All *Salmonella* spp. tested were found sensitive to colistin while 26.7% of *E. coli* spp. were colistin resistant. Contamination of chicken litter may be an underestimated source of antimicrobial resistance (AMR) transmission towards animals, humans and the environment with multidrug resistant *E. coli* and *Salmonella* spp. This shows the need and opportunity for a One Health approach in AMR surveillance and control in Cameroon. Continued surveillance in chicken litter would enable monitoring of AMR risks and trends.

## 1. Introduction

Poultry litter is a mixture of feces, wasted feeds, bedding material and feathers. It is a rich organic and cheap soil fertilizer that improves crop quality and productivity, hence explaining its widespread use as manure worldwide [1]. With the expansion of the poultry industry in all regions across the world, production of poultry litter as a waste product has also increased, further encouraging its use as manure. However, besides its organic content, poultry litter can be contaminated with various types of pathogens including viruses, bacteria, parasites and fungi. Foodborne bacteria such as *E. coli, Salmonella* and *Campylobacter* spp. have been isolated in poultry litter [2]; these bacteria pose a risk of transmission to animals, humans and the environment; especially considering their ability to survive for months in water, soil and crops [3,4]. Besides the risk of microbial contamination, there is an additional concern of transmission of multidrug resistant bacteria, due to the reported high use of antibiotics in poultry production either as growth promoters [5] or for prophylactic purposes [6]. Cameroon’s livestock production and agricultural subsistence farming practices have intensified in recent decades. The poultry sector specifically, has expanded since 2005 when restrictions on import of frozen chicken were introduced [7]. Local production consists mainly of broiler chicken production, from which the resulting chicken litter is the main manure used in the country. So far, little is known of the microbiological safety patterns and antimicrobial resistance (AMR) threat in livestock and production in Cameroon. Previous studies in farms and farmers showed presence of bacteria in chicken meat and other products [8], but not in chicken manure. 

This study was conceived as a pilot to explore options for integrated surveillance of AMR in foodborne bacteria in line with the WHO/OIE/FAO joint recommendation [9]. We estimated the prevalence of *E. coli* and *Salmonella* spp. in chicken litter in urban farming in Cameroon, and calculated the proportion of AMR in the isolated bacteria. We also assessed the use of antibiotics by farmers, using the AWaRE (ACCESS, WATCH and RESERVE) WHO classification [10]. Thereby, we aimed to establish if continued expanded surveillance in chicken litter would be feasible and useful as a One Health surveillance of AMR.

## 2. Results

### 2.1. General Characteristics of Farms

A total of 26 farms were visited and 71 samples of poultry litter collected (median number of samples/farm 2, interquartile range (IQR) (2–6). Mean age of farmers was 38 ± 11 SD years with a male predominance: 62%. The median duration of farming activities was 7 years (range 1–33 years). Only two farmers reported to have received initial training from an official organization before getting involved into the farming production activity. No farmers reported to have increased health concerns since they practiced farming activities. Most farms were semi intensive farms raising broiler chicken for commercial purposes. The majority of farms were situated within the household compound. Almost half of farmers (46%) decontaminated wood shavings in between batches of poultry, but no farmer reported decontaminating litter prior to disposal. All farmers reported to have access to veterinary services and reported to procure their medications in veterinary pharmacies. Only 38% of them systematically used these services whereas 24% never used them. A summary of other characteristics is presented in Table 1.

### 2.2. Antibiotic Use in Farms

**Figure 1:**
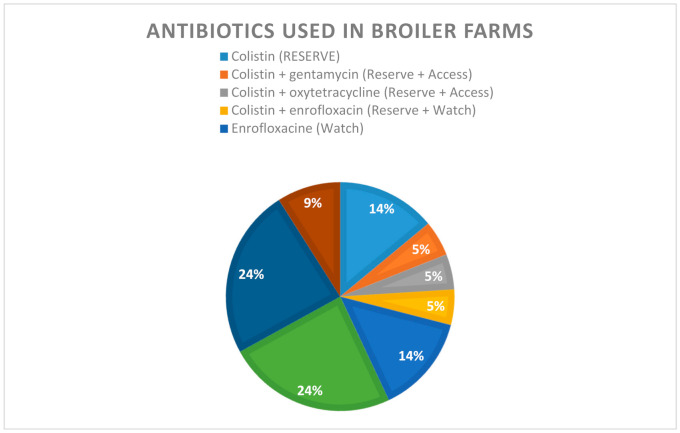
Antibiotics used in broiler chicken farms and their WHO AWaRe category.

Antibiotics given at farms belonged to the polymyxin, quinolones, and tetracycline and sulphonamides families. There were also farmers who gave combinations of antibiotics and very few who did not give antibiotics at all. Assessment of knowledge of farmers showed that 31% (8/26) of farmers could not give an appropriate name of an antibiotic used in poultry production. In broiler farms, 90% (19/21) of farmers used prophylactic antibiotics, whereas in layer and traditional farms, antibiotics were used for curative purposes only. More than 40% of farmers in broiler farms gave antibiotics for prophylactic purposes twice over a period of 45 days of the chicken’s production. Nearly 18% of these farmers gave prophylactic antibiotics four times within the same period of time. Quinolones (enrofloxacin and norfloxacin) were the most frequently used antibiotics (38%), followed by oxytetracycline (24%) and colistin (14%). About 15% of farmers gave combinations of two different classes of antibiotics, all including colistin (Figure 1). In our study, the largest group of antibiotics used belonged to the WATCH category (38%), whereas 33% and 14% fell under the ACCESS and the RESERVE categories, respectively. A smaller group (5%) belonged to a mix of ACCESS and RESERVE antibiotics.

### 2.3. Prevalence of E. coli and Salmonella spp.

*E. coli* spp. were isolated in 80.8% of farms and *Salmonella* spp. in 36.8% of farms. Out of the 71 samples collected, 45 were collected in house and 26 in stored bags ready to be used as manure. The proportion of isolation into bags and in door litter did not significantly vary for the two pathogens. Prevalence did not differ according to the location of sampling either, as reflected in Table 2.

### 2.4. Susceptibility and Resistance Patterns of E. coli and Salmonella spp.

**Figure 2:**
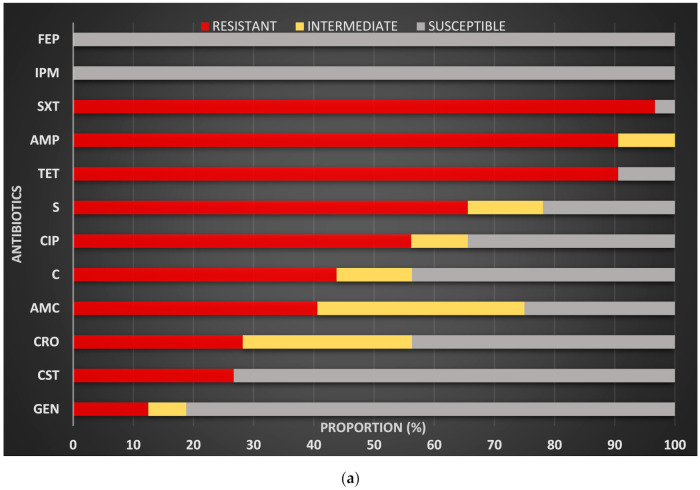

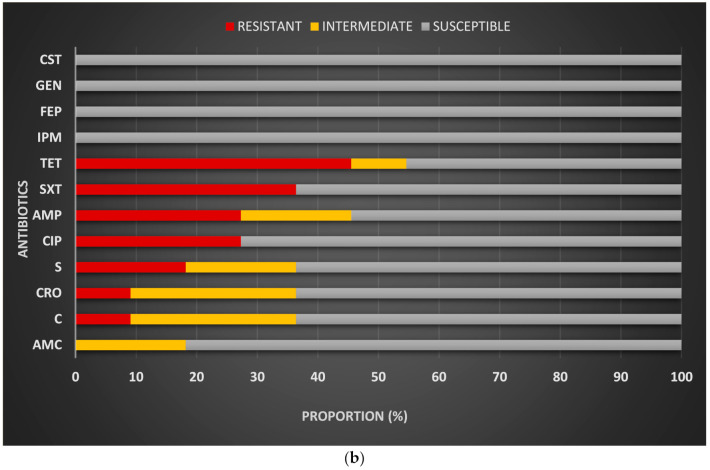
(**a**) Susceptibility and resistance patterns of *E. coli* spp. isolated from chicken litter; (**b**) Susceptibility and resistance patterns of *Salmonella* spp isolated from chicken litter. Legend: **AMC** (amoxicillin + clavulanic acid), **AMP** (ampicillin), **C** (Chloramphenicol), **CIP** (ciprofloxacin), **CRO** (cefrtiaxone), **CST** (colistin), **FEP** (cefepime), **GEN** (Gentamycin), **IPM** (Imipenem), **S** (strepromycin), **SXT** (trimethoprime + sulfamethoxazole), **TET** (tetracycline).

Out of the 12 antibiotics tested, highest resistance rates in *E. coli* were observed for trimethoprim + sulfamethoxazole, ampicillin, tetracycline and streptomycin. More than half of isolates tested were resistant to ciprofloxacin, a little more than a quarter were resistant to colistin, whereas low resistance was observed for gentamycin. No resistance was observed for cefepime and imipenem (Figure 2a). All the isolated *Salmonella* species tested were susceptible to imipenem, gentamycin, cefepime and colistin. For other antibiotics, resistance was observed with the highest frequencies for tetracycline, followed up by trimethoprim + sulfamethoxazole, ciprofloxacin and ampicillin (Figure 2b).

All *E. coli* isolates were multidrug resistant. One isolate was resistant to 9 out of the 11 antibiotics tested by the disc diffusion method. About 28% of *E. coli* isolates were resistant to five antibiotics or more. For *Salmonella* spp., 36% were multidrug resistant while 27% of isolates were found to be sensitive to all antibiotics tested. Co-resistance patterns for *E. coli* and *Salmonella* isolates are presented in Table 3.

Table 4 presents the number of *E. coli* and *Salmonella* spp. isolated according to the type of antibiotic given and from this, the proportion of resistant isolates for ciprofloxacin, tetracycline and trimethoprim + sulfamethoxazole was calculated.

In *E. coli* species, high resistance patterns were observed for tetracycline and trimethoprim + sulfamethoxazole, regardless of the type of antibiotics given at farms. In *Salmonella* species, no resistance to CIP, TET and SXT was observed out of the four isolates from farms receiving quinolones, whereas in farms receiving antibiotic combinations, three of four isolates were resistant to CIP, TET and SXT (Table 4).

### 2.5. Risks Factors for Salmonella Contamination

As *Salmonella* spp. are known to be less frequent than *E. coli* spp., we explored from our questionnaire potential risks factors for the presence of Salmonella species in our samples. However, we did not observe a statistically significant association between the size of the flock (*p* = 0.35), pre-treatment of litter (*p* = 0.72), the season (*p* = 0.11) and the presence of *Salmonella* spp. in poultry litter.

## 3. Discussion

### 3.1. Antibiotic Use

Of the antibiotics given for prophylactic purposes, almost half belonged to the WHO WATCH and RESERVE group. This is a major One Health concern, whereby it should be noted that WHO/FAO and OIE guidelines do not recommend prophylactic use of these categories of antibiotics in food producing animals. Quinolones, classified as WATCH drugs, were the most frequent antibiotics (38% of all) given at farms. These results were consistent with previous reports in the country, where around 30% and 57% of antibiotics used were quinolones [11]. Persistent use of quinolones for prophylactic purposes conducted over a three-year period of time shows low implementation of international recommendations and also demonstrates shortcomings in regulatory activities. Additionally, as quinolones are considered critically important antibiotics, their extensive use for prophylactic purposes in chicken production represents a serious threat that can contribute to spreading AMR throughout the poultry production chain.

We observed a very high proportion (>90%) of prophylactic antibiotic use compared to previous reports from Cameroon: 4% and 11% in 2015, respectively [11,12]. Different methods to assess use of antibiotics could be one explanation for the difference observed. Indeed, 31% of farmers could not give an appropriate name of antibiotics used, whereas, while crosschecking the types of products given, 90% of broiler farmers were giving antibiotics. Hence, assessment of the use of antibiotics by farmers, assuming sufficient knowledge may have biased previous results. Very high use of antibiotics, as observed in our study, could be explained by mistaken beliefs in the protective action of these drugs on livestock. This inappropriate behaviour may have increased following the 2016 avian influenza epidemic that occurred in the country, which caused high mortality rates in flocks and induced serious economic losses in the poultry industry [13]. This highlights the importance to assess behavioral changes that may occur among farmers following epidemics affecting the production system. 

Consequences of high prophylactic use of antibiotics can be discussed at various levels. In terms of health consequences, beside the global risk of emergence of multidrug resistant bacteria in poultry and indirectly in humans, there are also concerns linked to the presence of antibiotic residues in poultry products (meat and eggs). In Yaoundé, the capital city of Cameroon, Guetiya et al. detected high residual levels of chloramphenicol and tetracycline in chicken’s muscle [12]. Presence of these residues in tissues can not only drive resistance through suboptimal concentrations ingested, but it can also enhance allergic reactions in consumers, as it is the case for penicillin derivatives, or increasing the risk of abnormalities such as poor development of fetuses, staining of teeth in young children or gastro intestinal disorders from tetracycline residues [14]. As with environmental consequences, residues in the environment when litter is spilled will enhance development of multidrug resistant bacteria. As an example, Australian authors observed that environmental *Pseudomonas* spp. exposed to 1/10 of minimal inhibitory concentration of antibiotics developed genomic and phenotypic changes [15]. Further studies assessing the presence of antibiotic residues in soils, water and environment around poultry farms could provide additional key information about such contamination of the environment by the poultry production chain. 

High use of antibiotics in poultry production and its adverse consequences should sensitize the scientific community on the need to assess solutions that could decrease microbiological infection in flocks without increasing the risk of development of AMR. Essential oils and probiotics could be one of these alternatives. Different types of essential oils were tested either on ready to use products [16] or in vitro [17] and showed satisfactory antimicrobial properties. For probiotics, their uses have been shown to prevent occurrence of microbiological contamination while improving performances. However, effectiveness may depend on the type of probiotics used as well as internal and external factors [18].

In terms of procurement of antibiotics, all farmers reported to procure their medications at official veterinary pharmacies. This would avoid use of counterfeit or illegal drugs that may have an impact on their production. However, as less than a half of these farmers systematically used veterinary services, it is conceivable that a large part of delivery of medications at these pharmacies were done without prescription, raising concerns on the role of the need to conduct further assessment of delivery of antibiotics at these pharmacies, intensive sensitization of veterinary pharmacists and reinforcement of delivery regulations. 

### 3.2. Antibiotic Resistance

OIE, FAO and WHO have recommended an integrated and regular monitoring of foodborne pathogens, mainly *E coli*, *Salmonella* and *Campylobacter* spp. in food production systems [9]. Our study focused on prevalence and resistance patterns of *E. coli* and *Salmonella* spp., as they are reported to frequently contaminate chicken litter and they are easy to isolate. We observed that about 60% of samples and 80% of farms were contaminated with *E. coli* spp. High contamination with *E. coli* spp. is not surprising, as they are naturally colonizing the intestine of poultry and can contaminate litter via feces. We found higher contamination with *E. coli* spp. in studies where two selective media were used for isolation of the species [19] and comparable prevalence to studies which used only one selective media as we did [20]. This suggests that adding an additional selective media for the identification of *E. coli* species may increase the sensitivity of detection and we recommend for further studies a systematic use of two selective media. Antimicrobial susceptibility testing for *E. coli* isolates showed high resistance patterns to trimethoprim + sulfamethoxazole, ampicillin, tetracycline, streptomycin and ciprofloxacin. Our findings aligned with other reports, and this resistance pattern may result from selective pressure induced by the use of these antibiotics [6]. Despite high use of colistin as prophylaxis in broiler farms, we did not find resistant *Salmonella* spp. to colistin but more than a quarter of colistin resistance in *E. coli* spp., (although due to technical challenges we were not able to test all isolates for colistin susceptibility). Some authors observed similar patterns of colistin resistance: few or no resistance of *Salmonella* species; and *E. coli* spp. resistance between 18–26% [21,22,23]. In our study, absence of resistance in Salmonella species can be explained by our low sample size, whereas the proportion of resistance found in *E. coli* species represents an additional alarm bell for the monitoring of use of antibiotics as well as the surveillance of resistance in food producing farms.

We found high resistance to streptomycin compared to low resistance to gentamycin, although both molecules are aminoglycosides. Other authors found similar patterns in *E. coli* spp. [24] and suggested that this could be due to the fact that streptomycin are older molecules than gentamycin, with a higher risk of development of resistance. We did not observe phenotypic resistance to cefepime and imipenem, which could be explained by the fact that use of cephalosporin and carbapenems has not been reported in poultry production in Cameroon. Therefore, there has been no selective pressure on these antibiotics, reducing the risk of development of resistance. This suggests that multidrug resistance observed in our study may be linked to overuse of common antibiotics in poultry farms and it highlights the importance to perform regular monitoring of antibiotic uses in animal production systems, as recommended by WHO, OIE and FAO [9]. 

Compared to *E. coli* spp., which frequently contaminate chicken litter, contamination with *Salmonella* spp. is less frequent and appears to be enhanced by factors such as rainy season, reuse of litter for consecutive flocks or contamination of the flock with the pathogen [25]. Our prevalence of *Salmonella* spp. in chicken litter (15.5% of samples) was close to those of Tabo et al. in Chad [26] and Shang et al. in South Korea [27], with a prevalence of 15.6% and 11.1%, respectively. We could not identify a significant association with the season, the size of the flock nor pre-treatment of litter for contamination of chicken litter with *Salmonella* spp., although lack of significance could be due to a small sample size. Antibiotic susceptibility testing of *Salmonella* spp. isolates found higher resistance patterns for tetracycline, trimethoprim + sulfamethoxazole, but no phenotypic resistance to imipenem, gentamycin or cefepime. Nevertheless, absence of phenotypic resistance does not exclude the presence of genotypic mutations and further molecular testing is therefore recommended. Overall, our findings align with those of Abunna et al. in Ethiopia, who observed no resistance to gentamycin either but high resistance to tetracycline [28].

As described in other studies, *E. coli* spp. were found to be highly multidrug resistant, whereas *Salmonella* spp. were overall more susceptible to the antibiotics tested. This can be explained by the fact that *E. coli* are commensal pathogens of the poultry gut and they are more susceptible to antibiotic selective pressure and therefore development of resistance. Meanwhile, for *Salmonella* spp., it has been suggested that Gram-positive bacteria tend to acquire resistance genes from the resident bacteria in their environment (usually gram positive bacteria) and acquisition is influenced by the abundance of the resistance reservoir [29]. As *E. coli* spp. are considered to be resistance genes reservoirs, multidrug resistant *E. coli* found in our study indicates the risk of spread of resistance genes to other bacteria and enhancement of AMR. It would therefore be relevant in future studies to assess transmission of these resistance genes to other bacteria present in the environment. Additionally, as these bacteria can persist for several months in the environment, assessment of their presence and persistence on crops and soils following use of litter as manure will be relevant as this can reveal a hidden One Health threat, especially in these cases where no decontamination is performed prior to disposal of litter. 

As further perspectives for this study, molecular assays will be performed to look for resistance genes in the isolates, including β lactamase and colistin resistance genes. Results of this additional research will be part of another publication.

## 4. Materials and Methods 

### 4.1. Study Site and Population

The study was conducted in the capital city Yaoundé from December 2018 until March 2019. A list of eligible farmers with their contact details were obtained from the regional services for fisheries, animal industries and husbandries (Reference N°000176/L/MINEPIA/SG/DREPIA-CE). Additional farmers were included on recommendation by their peers.

### 4.2. Inclusion Criteria

Appointments were booked by phone with farm owners to plan a site visit. On site, following oral explanations and after obtaining informed consent, a short questionnaire assessing farming practices, including use of antibiotics was administered. 

### 4.3. Samples Collection and Processing

Samples of chicken litter were collected from both buildings and storage bags (when available) at each participating farm. Using sterile gloves, litter was mixed and collected at different places of the building or the bag. Small quantities collected were added into a sterile 100 mL plastic container until full. A code was attributed to each container and samples were placed in a cooler containing ice packs prior to the transfer within four hours to the National Veterinary Laboratory (LANAVET- Yaoundé Branch) and LABOREB where analyses were performed.

### 4.4. Microbiological Assays

Pre-enrichment suspension was obtained by adding 25 mg of poultry litter into 225 mL of buffered peptone water, which were incubated at 35 ± 2 °C for 16–24 h. Isolation of *E. coli* spp. was done by plating pre-enrichment suspension on McConkey agar followed by incubation. Suspect lactose-positive colonies on McConkey agar were submitted to biochemical tests using a mini gallery. Isolation and identification of *Salmonella* spp. were performed following ISO 6579:2002 recommendations and confirmation was done using biochemical tests in mini gallery and API 20E (Biomérieux, Lyon, France). 

### 4.5. Susceptibility Testing

A panel of 12 antibiotics frequently used in human medicine in Cameroon was selected and tested on the isolates. For both species, antibiotic susceptibility testing was done using the disc diffusion method except for colistin susceptibility testing, a microbroth dilution method was used [30]. A picture of a colistin microbroth dilution plate performed on the isolates is available as a Supplementary Materials.

CLSI standards [31] were used to classify susceptibility of isolates.

### 4.6. Data Collation and Analysis

Data and information from each farm and broilers were collated and entered into an Excel spread sheet. Data quality was checked by independent assessors. Assessment of knowledge of what an antibiotic is was done by asking the farmer to give an appropriate name of an antibiotic (either active principle or brand name). Antibiotics used were grouped into 3 categories according to the WHO Access-Watch-Reserve classification [10]. The ACCESS category includes antibiotics that should be widely available, affordable and quality-assured. The WATCH category includes antibiotic classes that have higher resistance potential and so are recommended as first or second choice treatments only for a specific, limited number of indications. These medicines should be prioritized as key targets of stewardship programs and monitoring. This group includes most of the highest priority agents among the Critically Important Antimicrobials for Human Medicine and/or antibiotics that are at relatively high risk of selection of bacterial resistance [10]. The RESERVE group includes antibiotics that should be treated as “last resort” options that should be accessible, but whose use should be tailored to highly specific patients and settings, when all alternatives have failed. These medicines could be protected and prioritized as key targets of national and international stewardship programs involving monitoring and utilization reporting to preserve their effectiveness [10].

Data were analyzed with R packages.

### 4.7. Administrative Authorization

The authorization to conduct the study was obtained from the regional services for fisheries, animal industries and husbandries (Reference N°000176/L/MINEPIA/SG/DREPIA-CE).

## 5. Conclusions

Our study found high use of antibiotics for prophylactic purposes in broiler farms in Cameroon, including antibiotics listed as WATCH or RESERVE. This was enhanced by a lack of knowledge on antibiotics among farmers and a passive role of veterinary pharmacies from which medications could be purchased apparently without prescription. Overuse of antibiotics does not only favor the risk of emergence and transmission of multidrug resistant bacteria, but it also poses a problem of food and environmental contamination with antibiotic residues that can create severe threats for humans, animals and the environment. We isolated significant amounts of *E. coli* and *Salmonella* spp. from chicken litter and in particular, many of the *E. coli* spp. tested were multidrug resistant. As no treatment was performed to reduce microbial contamination of chicken litter prior to its use as manure, we can conclude that poultry litter can be a source of environmental contamination with multidrug resistant bacteria. This supports WHO/FAO/OIE recommendations for the setting up of integrated surveillance of AMR in key foodborne bacteria, and provided useful information for the Cameroon authorities in controlling AMR in the country. Establishing AMR surveillance in poultry litter could additionally strengthen prevention and control of AMR in Cameroon. 

## Figures and Tables

**Table 1:** General characteristics of farms.

Variable	Outcome
Type of farms	Semi intensive farms 96% (25/26) Traditional farms 4% (1/26)
Types of species breed	Broiler chicken 86% (21/26) Broiler and layer chicken 12% (13/26) Layer chicken 7% (2/26)
Size of the flock	Median size 1000 (10–6000)
Location of the farm	Within a household compound 69% (18/26) Outside an household compound 31% (8/26)
Number of people working on the farm	Mean 2.4 ± 1.3 (SD)
Number of people living near the farm	Mean 5.8 ± 4.7 (SD)
Food origin	Commercial feed mills 100% (26/26)
Type of bedding material used	Wood shavings 100% (26/26)
Decontamination of bedding material before use	Yes 46% (12/26) No 54% (14/26)
Mean duration of poultry litter prior disposal	Broiler farms 41 days Layer farms 324 days

**Table 2:** Prevalence of *E. coli* and *Salmonella* spp. in 71 poultry litter samples.

	*E. coli* spp.	*Salmonella* spp.
**Samples**	Prevalence	Prevalence
**In House Samples (N = 45)**	26 (57.8%)	7 (15.6%)
**Bags (N = 26)**	16 (61.5%)	4 (15.4%)
**Total**	59.2% (95% Confidence Interval-CI 46.8–70.5)	15.5% (95%CI 8.4–26.5)

**Table 3:** Co resistance patterns of *E. coli* and *Salmonella* spp. isolates.

Number of Antibiotics	Isolates	Antibiotic Resistance Pattern	Number of Isolates	Origin of Sample
**1**	*Salmonella* spp.	CRO	1	IN HOUSE
**2**	*Salmonella* spp.	C + TET	1	BAG
**3**	*E. coli* spp.	SXT + C + CIP	1	IN HOUSE
		SXT + TET + AMP	3	IN HOUSE, BAG
		AMP + CRO + C	1	IN HOUSE
		SXT + TET + STREP	2	IN HOUSE
	*Salmonella* spp.	TET + SXT + CIP	1	IN HOUSE
**4**	*E. coli* spp.	AMP + S + TET + SXT	2	IN HOUSE, BAG
		AMP + TET + SXT + CIP	1	IN HOUSE
	*Salmonella* spp.	AMP + TET + SXT + CIP	1	IN HOUSE
		AMP + S + TET + SXT	1	IN HOUSE
**5**	*E. coli* spp.	AMC + AMP + TET + SXT + CHL	3	IN HOUSE, BAG
		AMP + S + TET + SXT + CIPR	1	IN HOUSE
		AMP + CRO + S + SXT + CIPR	1	BAG
		AMP + AMC + CRO + TET + SXT	1	IN HOUSE
		AMP + AMC + S + TET + SXT	2	IN HOUSE
		GEN + S + TET + SXT + CIP	1	IN HOUSE
	*Salmonella* spp.	AMP + S + TET + SXT + CIP	1	IN HOUSE
**6**	*E. coli* spp.	AMP + S + TET + SXT + C + CIP	4	IN HOUSE, BAG
		AMC + AMP + CRO + S + TET + SXT	1	IN HOUSE
		AMC + AMP + S + TET + SXT + CIP	1	IN HOUSE
**7**	*E. coli* spp.	AMP + AMC + CRO + S + TET + SXT + CIP	2	IN HOUSE
		AMP + GEN + S + TET + SXT + C + CIP	1	IN HOUSE
		AMP + CRO + S + TET + SXT + C + CIP	1	BAG
		AMP + AMC + S + TET + SXT + C + CIP	1	IN HOUSE
		AMP + AMC + GEN + TET + SXT + C + CIP	1	IN HOUSE
**9**	*E. coli* spp.	AMP + AMC + CRO + S + TET + GEN + SXT + C + CIP	1	IN HOUSE

Legend: **AMC** (amoxicillin + clavulanic acid), **AMP** (ampicillin), **C** (Chloramphenicol), **CIP** (ciprofloxacin), **CRO** (ceftriaxone), **GEN** (Gentamycin), **IPM** (Imipenem), **S** (strepromycin), **SXT** (trimethoprime + sulfamethoxazole), **TET** (tetracycline).

**Table 4:** Antibiotics given at farms in relation to resistance patterns in *E. coli* and *Salmonella* spp.

		Resistance to CIP *E.coli* Isolates (%)	Resistance to TET *E.coli* Isolates (%)	Resistance to SXT *E.coli* Isolates (%)		Resistance to CIP *Salmonella* Isolates (%)	Resistance to Tet *Salmonella* Isolates (%)	Resistance to SXT *Salmonella* Isolates (%)
**Family of Antibiotics Given at Farm**	**Number of ** ***E. coli*** ** Isolates**				**Number of Salmonella Isolates**			
**Polymyxins**	**1**	100	100%	100%	**0**			
**Quinolones**	**13**	69	85%	100%	**4**	0	0	0
**Tetracyclin**	**12**	33	100%	100%	**1**	0	100	100
**Sulfonamides**	**3**	100	100%	100%	**2**	0	50	0
**Antibiotic Combinations**	**1**	0	100%	100%	**4**	75%	75	75
**No Antibiotic**	**2**	50%	50%	50%	**0**			

## Supplementary Materials

The following are available online at https://www.mdpi.com/2079-6382/10/1/20/s1. A picture of a colistin microbroth dilution plate performed on the isolates is available.
