# Supplementary material for: Microbial Contamination of Chicken Litter Manure and Antimicrobial Resistance Threat in an Urban Area Setting in Cameroon
Source: Antibiotics (Basel). 2020 Dec 29;10(1):20. doi: 10.3390/antibiotics10010020 (PMC7824223; doi:10.3390/antibiotics10010020)
Supplement: Supplementary file 1 [file antibiotics-10-00020-s001.pdf]

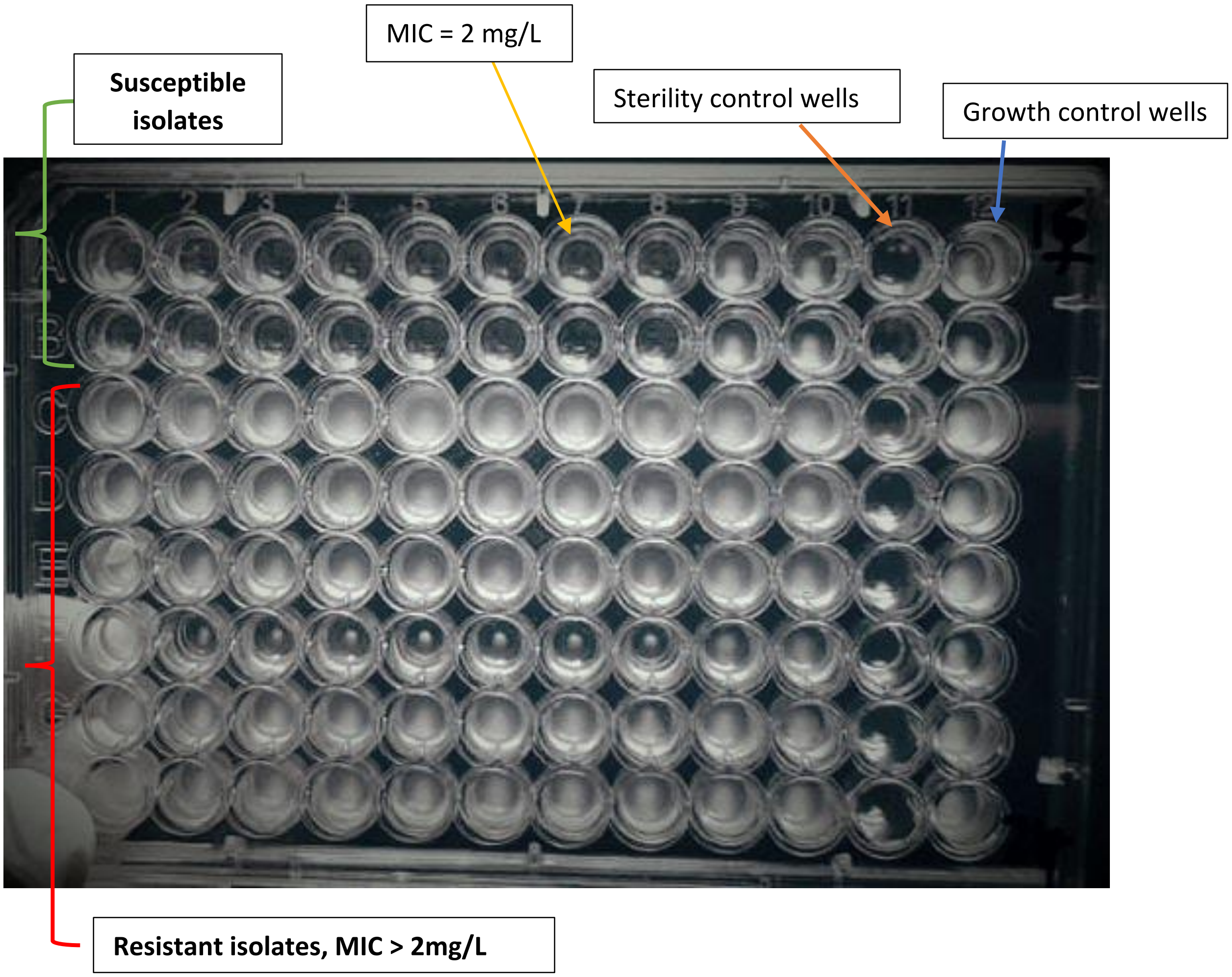

The picture shows a plate of colistin broth micro dilution assay performed on *E.coli spp* isolated from poultry litter. As part of the quality control for the assay, growth was effective in all the growth control wells (column 12) while no growth was observed in the sterility control well (column11). Antibiotics concentration varied from 0.25 (column10) – 128 (column1) mg/L. Isolates are considered resistant to colistin when the minimal inhibitory concentration (MIC) is equal to or greater than 2mg/L.
